# Prevalence of Metabolic Syndrome in Overweight and Obese Patients and Their Measurement of Neck Circumference: A Cross-sectional Study

**DOI:** 10.7759/cureus.6114

**Published:** 2019-11-10

**Authors:** Ayesha A Hai, Sundus Iftikhar, Saba Latif, Fivzia Herekar, Sana Javed, Muhammad Junaid Patel

**Affiliations:** 1 Internal Medicine, The Indus Hospital, Karachi, PAK; 2 Statistics, Indus Hospital Research Center, The Indus Hospital, Karachi, PAK; 3 Internal Medicine and Infectious Diseases, The Indus Hospital, Karachi, PAK

**Keywords:** metabolic syndrome, overweight, obese

## Abstract

Background

The utilization of neck circumference (NC) as a valuable tool to evaluate metabolic syndrome (MetS) is still unclear. MetS has been extensively reported worldwide mainly due to the increasing trend of central obesity and the risk of developing coronary artery disease. In Pakistan, its incidence is reported to be between 18% and 49% among the urban population.

Methods

This cross-sectional study was conducted at the Indus Hospital, Karachi, Pakistan; in total, 392 patients (body mass index [BMI] >23 kg/m^2^, age ≥18 years, both genders) were recruited through consecutive sampling, and informed consent was obtained.

Results

The majority of patients were females (*n *= 344, 87.8%), and the mean ± SD of age and NC of all patients was 50.5 ± 9.6 years and 38 ± 4.6 cm, respectively. The majority (*n* = 375, 95.7%) of patients were found to have MetS, with 90% of both males and females having NC ≥38 cm and 34 cm, respectively.

Conclusion

The prevalence of MetS was found to be very high in overweight and obese patients. Moreover, the majority of patients with MetS were found to have higher NC.

## Introduction

Globally, the burden of overweight and obesity has been increasing substantially affecting almost 30% of the entire world population [1]. Overweight and obesity are defined as an excessive accumulation of body fat or weight that exceeds the age- and gender-specific reference limits leading to ill health and a high incidence of non-communicable diseases such as metabolic syndrome (MetS) [1-2]. Pakistan ranks ninth among the world's most obese nations [1].

MetS is a serious health condition that can lead to several chronic, non-communicable diseases such as cardiovascular disease and diabetes mellitus [3]. MetS is a combination of at least three of the following five metabolic abnormalities: 1) elevated waist circumference (cutoff for Asians: ≥90 cm/35.4 inches in men and ≥80 cm/31.5 inches in women), 2) elevated triglycerides (TG; ≥150 mg/dL), 3) reduced high-density lipoprotein (HDL; <40 mg/dL in men and <50 mg/dL in women), 4) elevated blood pressure (systolic blood pressure [SBP] ≥130 mmHg or diastolic blood pressure [DBP] ≥85 mm Hg) or hypertensive, and 5) elevated fasting blood glucose level (≥100 mg/dL or 5.6 mmol/L) or diabetic [4].

There are several criteria for diagnosing MetS, of which the National Cholesterol Education Program Adult Treatment Panel III (NCEP ATP III) and the International Diabetes Federation (IDF) are the most widely used criteria in Pakistan due to their reliability [5-6]. According to these criteria, the prevalence of MetS in Pakistan over the last decade is reported to be 35% to 64% [7-8]. According to the report of the Global Burden of Disease (GBD), the disability-adjusted life years (DALYs) attributable to risk factors associated with the diagnosis of MetS have dramatically increased over the past two decades [9]. The report revealed that in 2010, high BMI and diabetes ranked sixth among the various leading risk factors and non-communicable diseases contributing to DALYs [9].

Waist circumference (WC) is commonly used to define central obesity and plays a vital role in diagnosing obesity and MetS. The measurement of WC may be significantly influenced by various measuring methods, weather conditions, particularly winter, and interday variations that might affect the abdominal wall and abdominal cavity [10]. Literature has shown that the heterogeneity in body structure and abdominal tissues depend on not only age and gender but also ethnicity [11]. Hence, the IDF has now proposed lower cut-off values for WC for some ethnicities [11]. Moreover, BMI, which is considered as another valuable tool to assess overweight and obesity, does not evaluate fat distribution; hence, it cannot always assess the individual risk of endocrine and metabolic complications.

Therefore, several studies have been carried out to assess the alternative anthropometric measure for diagnosing overweight, obesity, and MetS. Recently, researchers have greatly focused on NC, a parameter of upper-body adiposity. NC is a simpler, relatively new and practical anthropometric indicator that is not affected by the postprandial abdominal distension or by respiratory movements and can be particularly useful in specific populations such as morbidly obese people, patients in bed rest, and pregnant women [9].

NC has been shown to be promising in diagnosing overweight and obesity and is also significantly associated with cardiovascular disease, SBP and DBP, and TG, total cholesterol, and fasting blood sugar levels; NC is also a predictor of obstructive sleep apnea syndrome in the Framingham study [7,12-13].

Moreover, recent studies have also reported a significant and positive correlation between NC and MetS [14-15]. A study done in Pakistan in 2016 has reported a higher NC in the MetS group in comparison to the non-MetS group [14]. Additionally, the study reported NC ≥38 cm and ≥34 cm as the optimal cutoff values for the diagnosis of MetS in males and females, respectively [14]. However, a systematic review and meta-analysis done in 2018 reported significant positive associations between NC and the various factors of MetS but has reported no association between the two [16].

We aimed to conduct this study to assess the prevalence of MetS owing to the dearth of local literature reporting the current burden of MetS in Pakistan. Also, this study intended to gauge the optimal cutoff values of NC provided by Hingorjo MR et al. [17].

## Materials and methods

This cross-sectional study was conducted at the Indus Hospital, Karachi (TIH), a free-of-cost tertiary care facility. A priori sample size was calculated using OpenEpi software (Open Source Epidemiologic Statistics for Public Health, Version 3.01) with the following assumptions: MetS prevalence of 49% and the desired precision of 5% [7]. The required sample size was estimated to be 384. All patients of either gender and age ≥18 years presenting to the outpatient clinics or admitted in the medical unit with BMI >23 kg/m^2 ^ as per the Asian criteria and had given informed consent were included in the study through non-probability consecutive sampling [18].

Patients with clinical or laboratory evidence of cardiac failure (stage C and D), liver failure, hyper or hypothyroidism, known congenital syndromes that lead to obesity, or on systemic steroids were excluded from the study.

Data on socio-demographic characteristics, such as age, gender, comorbidities, anthropometric variables (height, weight, BMI, NC, WC), and blood pressure, were gathered. MetS screening tool used by the Department of Mental Health, Missouri was used to assess MetS [17]. Recent biochemical profiles of individual patients such as fasting plasma glucose, TC, LDP, TG, and HDP levels were extracted from the Hospital Information Management System (HIMS) of TIH. All data were recorded on a pre-designed questionnaire.

Blood pressure was measured using the auscultatory method, which uses a mercury sphygmomanometer and stethoscope for determining the SBP and DBP based on the appearance and disappearance of Korotkoff sounds, respectively. Two readings for SBP and DBP were taken and their average value was used in the analysis. Anthropometric measurements were measured using a soft flexible measuring tape. WC was measured in centimeters at the midpoint between the lowest rib and the highest point of the iliac crest. NC was measured at the level of the lower part of thyroid cartilage (just below Adam’s apple) along a horizontal line in centimeters, with readings up to 1 decimal place.

Data analysis procedure

Data were entered on REDCap software (RedCap v9.1.0, Vanderbilt University) and analyzed using SPSS version 24.0 (IBM Corp, Armonk, NY). All the quantitative variables such as age, SBP and DBP, and biochemical parameters were assessed for normality and reported as mean ± SD or median (interquartile range [IQR]) as appropriate. The categorical variables were reported as frequencies and percentages. The prevalence of MetS was calculated using the criteria. Pearson or Kendall Tau-b correlation were used as appropriate to assess the relationship between NC and components of MetS. *P*-value <0.05 was considered significant.

## Results

A total of 392 patients were included in the study, and the majority of patients 344 (87.8%)were females. The mean ± SD age and NC of all the patients were 50.5 ± 9.6 years and 38 ± 4.6 cm, respectively (Table 1).

**Table 1:** Characteristics of the study participants IHD, ischemic heart disease

	n (%)
Gender	
Male	48 (12.2)
Female	344 (87.8)
Total	392 (100)
Ethnicity	
Sindhi	26 (6.6)
Balochi	8 (2)
Urdu speaking	185 (47.2)
Punjabi	72 (18.4)
Pathan	35 (8.9)
Others	66 (16.8)
Total	392 (100)
Occupation	
Professional	11 (2.8)
Technicians and associate professionals	1 (0.3)
Clerical support workers	8 (2)
Service and sales workers	3 (0.8)
Skilled agricultural, forestry and fishery workers	2 (0.5)
Craft and related trades workers	3 (0.8)
Housewife	303 (77.3)
Others	61 (15.6)
Total	392 (100)
Comorbids	
Hypertensive	309 (84.4)
IHD	31 (8.5)
Dyslipidemia	224 (61.2)
Diabetes	300 (82)
Others	135 (36.9)
Metabolic syndrome	
Non-metabolic syndrome	12 (3.1)
Metabolic syndrome	366 (93.4)
Unknown	14 (3.6)

More than three-quarters of the patients (n = 303; 77.3%) were homemakers, while 89 (22.7%) patients were doing any kind of job. Furthermore, comorbidities were present in all the patients. The most common comorbidity was hypertension (n = 309; 84.4%) followed by diabetes (n = 300; 82%) and dyslipidemia (n = 224; 61.2%).

Moreover, results showed that 366 (93.4%) of the patients has MetS, 12 (3.1%) were non-MetS, and 14 (3.6%) had unknown status due to incomplete information about the components of MetS (Table 2).

**Table 2:** Characteristics of the study participants with and without metabolic syndrome SD, standard deviation; IQR, interquartile range; IHD, ischemic heart disease, HDL, high-density lipoprotein; HbA1c, glycated hemoglobin

Characteristics	Metabolic syndrome		Overall	P-value
	No (n = 7)	Yes (n = 375)		
Age (in years)				
Mean ± SD	47.2 ± 13.5	50.5 ± 9.5	50.5 ± 9.6	0.749^ⱡ^
Min-Max	30 to 60	24 to 79	24 to 79	
Median (IQR)	55.5 (33 to 60)	50 (45 to 56)	50 (45 to 56)	
Height (cm)				
Mean ± SD	156.7 ± 7	154 ± 7.9	154.1 ± 7.9	0.249^ⱡ^
Min-Max	145 to 167.6	103 to 180	103 to 180	
Median (IQR)	157.5 (152.5 to 160)	153 (149.5 to 158)	153 (150 to 158.8)	
Weight (kg)				
Mean ± SD	78.8 ± 13.7	77.5 ± 13.4	77.6 ± 13.5	0.571^ⱡ^
Min-Max	60.4 to 95	50 to 130	50 to 130	
Median (IQR)	80 (61.5 to 91)	75 (68 to 84)	75 (68 to 84.9)	
BMI				
Mean ± SD	32 ± 4.6	32.7 ± 5.7	32.8 ± 5.7	0.946^ⱡ^
Min-Max	25.1 to 37.6	23.2 to 75.4	23.2 to 75.4	
Median (IQR)	33.1 (28.5 to 36.7)	31.9 (29.1 to 35.2)	31.9 (29.1 to 35.3)	
Waist circumference (cm)				
Mean ± SD	39.8 ± 4.5	43 ± 7	43.2 ± 7.7	0.080^ⱡ^
Min-Max	36 to 47	32 to 142	32 to 142	
Median (IQR)	38.8 (36 to 45)	42.3 (40 to 45)	42.2 (40 to 45)	
Neck circumference (cm)				
Mean ± SD	37.2 ± 2.8	37.9 ± 3.9	38 ± 4.6	0.667^ⱡ^
Min-Max	33 to 40.6	29 to 78.7	29 to 88.9	
Median (IQR)	36.8 (35.6 to 40.6)	38.1 (35.6 to 40.6)	38.1 (35.6 to 40.6)	
Systolic blood pressure (mm of Hg)				
Mean ± SD	115.3 ± 9.6	129.6 ± 16.6	129.3 ± 16.5	0.009^*^^ⱡ^
Min-Max	100 to 130	90 to 190	90 to 190	
Median (IQR)	117 (110 to 120)	130 (120 to 140)	130 (120 to 140)	
Diastolic blood pressure (mm of Hg)				
Mean ± SD	71.4 ± 3.8	78.8 ± 10.8	78.6 ± 10.7	0.038^*^^ⱡ^
Min-Max	70 to 80	60 to 110	60 to 110	
Median (IQR)	70 (70 to 70)	80 (70 to 90)	80 (70 to 90)	
Fasting blood glucose				
Mean ± SD	96.9 ± 15.7	147.8 ± 61.3	145.8 ± 61	0.001^*^^ⱡ^
Min-Max	87 to 130	43 to 438	43 to 438	
Median (IQR)	90 (87 to 104)	132 (108 to 167.3)	129.5 (106 to 165.5)	
Total cholestrol				
Mean ± SD	185.4 ± 36.7	167.3 ± 44.1	167.8 ± 43.6	0.281^†^
Min-Max	129 to 226	53 to 314.1	53 to 314.1	
Median (IQR)	197 (138.7 to 206)	166 (135 to 196)	166.2 (136 to 197)	
Triglyceride				
Mean ± SD	90.3 ± 28.4	167.7 ± 81.1	164.9 ± 80.8	0.001^*^^ⱡ^
Min-Max	65 to 135	27 to 604.7	27 to 604.7	
Median (IQR)	80 (65 to 118)	150 (116.3 to 195.8)	147 (115 to 193.5)	
HDL				
Mean ± SD	55 ± 11.7	38.5 ± 9.7	39.1 ± 10.3	0.001^*^^ⱡ^
Min-Max	41 to 69.8	14 to 91	14 to 91	
Median (IQR)	57 (42.3 to 69)	37.1 (32 to 43.5)	38 (32.5 to 44)	
LDL				
Mean ± SD	119.3 ± 35.5	110.1 ± 38.6	110.3 ± 38.2	0.398^ⱡ^
Min-Max	63 to 157	25 to 228	25 - 228	
Median (IQR)	128 (78.6 - 151)	105 (81 to 137.7)	106 (81.2 to 137)	
VLDL-Cholesterol				
Mean ± SD	18 ± 5.8	34 ± 18.5	33.4 ± 18.4	0.001^*^^ⱡ^
Min-Max	13 to 27	5 to 206	5 to 206	
Median (IQR)	16 (13 to 24)	30 (23 to 39.3)	29 (23 to 39)	
Non-HDL Cholesterol				
Mean ± SD	130.4 ± 40	129.1 ± 42.2	128.8 ± 41.9	0.736^ⱡ^
Min-Max	69 to 169	35 to 273	35 to 273	
Median (IQR)	140 (80 to 162)	127 (97 to 155.3)	127.5 (97 to 155.2)	
HbA1c				
Mean ± SD	5.4 ± 0.6	8 ± 2	7.9 ± 2	0.000^**^^ⱡ^
Min-Max	4.6 to 6.3	4.1 to 14.9	4.1 to 14.9	
Median (IQR)	5.4 (4.8 to 6)	7.5 (6.4 to 9.2)	7.5 (6.3 to 9.1)	
Comorbid				
Hypertensive	2 (28.6)	316(84.3)^a^	324 (82.7)	0.000^**ɫ^
IHD	0(0)	32 (8.5)	33 (8.4)	
Dyslipidemia	6 (85.7)	224(60.3)	233 (59.4)	
Diabetes	1 (14.3)	303(80.8)^a^	304 (77.6)	
Others	5 (71.4)	141(37.6)	154 (39.3)	
*P-value<0.05, **P-value<0.0001, † Independent Sample T-test, ⱡ Mann-Whitney U test, ɫ Chi-square test				

It was observed that 90% of both males and females had NC ≥ 38 cm and 34 cm, respectively, and the optimal cutoff values were provided by Hingorjo MR et al. [16]. Also, females had lower NC than males (mean ± SD: 37.5 ± 3.7 cm vs 41.4 ± 3.2 cm, p < 0.0001).

Additionally, no significant linear correlation was between NC and HbA1C, total cholesterol, LDL, non-HDL cholesterol, fasting blood glucose levels, and SBP and DBP. A significant weak positive correlation was observed between NC and WC, VLDL cholesterol, and TG; however, a significant weak negative correlation was observed between NC and female gender and HDL (Table 3).

**Table 3:** Correlation of neck circumference with various variables BMI, body mass index; TG, triglyceride; HDL, high-density lipoprotein; VLDL, very low density lipoprotein; LDL, low-density lipoprotein

	Unadjusted correlation coefficient	P-value	Adjusted correlation coefficient
Females	-0.329	0.000^**^	-
BMI	0.184	0.000^**^	0.201**
Waist circumference in cm	0.290	0.000^**^	0.293**
TG	0.107	0.020^*^	0.112
HDL	-0.123	0.008^*^	-0.087
VLDL cholesterol	0.106	0.023^*^	0.109
HBA_RESULT	0.069	0.138	0.088
Total cholesterol	-0.068	0.140	-0.051
LDL	-0.069	0.134	-0.058
Non-HDL cholesterol	-0.039	0.394	-0.030
Fasting blood glucose	0.037	0.422	0.059
Systolic (mm of Hg)	0.019	0.695	0.011
Diastolic (mm of Hg)	0.002	0.966	-0.012
*P-value<0.05, **P-value<0.0001, Kendall Tau-b correlation			

On the contrary, NC was found to be significantly positively correlated with BMI and WC only adjusting for gender (Table 3).

## Discussion

The National Nutrition Health Survey (2018) of Pakistan reported a high prevalence of overweight and obesity in adolescents, young adults, and women of reproductive age [19]. Moreover, the prevalence of MetS has increased two folds over the last 10 years in Pakistan, indicating that these two are the emerging public health issues in Pakistan that require urgent attention of healthcare providers to restrain their increasing incidence [7-8]. Although Pakistan is facing an epidemic of obesity and MetS, no recent literature is available assessing the burden of MetS in general as well as in the overweight and obese population.

In this study, we found a very high prevalence of MetS (95.7%) in our overweight and obese patients in comparison to the previous studies done in Pakistan [5,7-8,20]. Also, in our study, the majority of patients were diabetic and hypertensive, which is one of the diagnostic components of MetS. Ahmed A et al. (2012) reported 91.9% prevalence of MetS in type 2 diabetic patients, whereas other studies reported a high prevalence of overweight and obesity among MetS patients [5,7,19]. Moreover, a wide variation in the prevalence of MetS has been reported in various developing countries, i.e., from 7.9% to 39% [21]. This enormous heterogeneity in the prevalence could be due to the different criteria used for MetS, ethnic variations, genetics, environmental and cultural differences, study settings, and other risk factors. Also, patients presenting to the Indus Hospital belong to low- and middle socioeconomic groups that could account for the high prevalence of MetS. Literature reports an association between low socioeconomic status (SES) and MetS as people belonging to low SES are less educated, have low healthcare knowledge, and less leisure-time physical activity and may be unemployed [22-23].

Furthermore, our study revealed that more than 90% of our study participants had NC greater than or equal to the optimal cut-off values provided by Hingorjo MR et al. [17]. Thus, this indicates that these cut-off values can be used to screen MetS in the Pakistani population, thereby reducing the diagnostic testing. Several studies have reported the use of NC for identifying obesity and MetS by various criteria in different populations [10,15,18,24-25].

Additionally, we found a significant positive association between NC and VLDL and TG. This result is also supported by a recent systematic review and meta-analysis [16]. Some of the components of MetS in our study were not significantly correlated with NC, which include HbA1c, SBP and DBP, TC, LDL, non-HDL, and fasting glucose levels, whereas Bochaliya et al., Cho et al., Ben-Noun, and Ataie Jafari showed significant association of these aforementioned variables with NC [23,26-28]. It was also shown by Ravikiran et al. that the risk of cardiovascular diseases and MetS among South Asians increased due to an increase in fat mass of intra-abdomen, body, and truncal subcutaneous and ectopic deposition, such as the region of the neck [29]. In our study, BMI and WC were significantly associated with NC. The significant association between NC and BMI and WC was observed in other studies as well, which identified NC as an efficient tool for the identification of patients with obesity and MetS [10,30]. Therefore, our study is in agreement with other studies that suggest NC as a good predictor of obesity and MetS.

Limitations

Since this study was a cross-sectional study, we could not establish an association between MetS and NC. We suggest either a longitudinal or a case-control study to establish any relationship between MetS and NC. Secondly, this is a single-center, hospital-based study with the majority of patients being females. Thirdly, in this study, we have included all the overweight and obese patients, out of which almost two-thirds were both diabetic and hypertensive, which are the components contributing to MetS due to which we found a very high prevalence of MetS. We recommend further community-based studies to assess the burden of the disease.

## Conclusions

A very high prevalence of MetS was observed among overweight and obese patients in this study. Moreover, NC can be used as a screening tool for MetS.
